# Varices

**Published:** 1875-05

**Authors:** H. H. Toland

**Affiliations:** San Francisco


### VARICES.

BY H. H. TOLAND, M. D., SAN FRANCISCO.

This is an exceedingly common disease, and consists of a thickening and dilatation of the coats of the veins. This difficulty occurs in the veins of the legs, in the hemorrhoidal, the spermatic, as well as in many of the superficial veins of the body—as the abdominal, when the vena cava is obstructed. When a hereditary predisposition exists, the veins of the leg may enlarge by the use of tight garters, by the pressure of the gravid uterus, and by remaining too long in the erect position. Whatever hinders or retards the circulation of the blood in the veins produces a dilatation, and generally a thickening of the coats as well as an extraordinary elongation. In a short time, in consequence of the irritation of the skin produced by the pressure of the enlarged veins, eczema appears, which is always accompanied by so much itching that the patient frequently tears off the skin and establishes an ulcer which cannot be cured without removing the cause.

These ulcers are called varicose, and, as no inconvenience results from the obliteration of the superficial veins, they are curable. The circulation is re-established by the dilatation of other branches. Even when the inferior vena cava is obliterated, the lower portion of the vessel as well as its branches dilate, such as the superficial abdominal or azygos, which carry the blood to the heart. When veins enlarge without supplying the place of some obliterated vessel or vessels, they are varicose, and not only useless but hurtful. As early as 1532 the disease was recognized and described by Paulus Ægineta.

True varices are generally found in the bladder, rectum, spermatic chord, and the lower extremities. The deep-seated veins often become dilated before the superficial enlarge and produce pains in the legs, as well as a want of power, produced by the pressure made upon the nerves by which the parts are supplied. Certain conditions of the system favor the production of this disease.

As already mentioned, the coats of the dilated vein generally become thickened, greatly elongated, and consequently tortuous. So soon as the valve cusps lose their power, the dilatation and elongation increase very rapidly, which is soon followed by eczema and ulceration. The feet are generally swollen and cold, particularly if the patient remains long in an erect position.

The internal saphena vein is more frequently affected than the external; occasionally the dilatation of this vein is irregular, and the coats become so thinned by the distension that they yield to the weight of the column of blood, which may cause a fatal hemorrhage. Sometimes the vessels become so distended that the blood coagulates, which obliterates the vessel permanently and effects a radical cure. When the distention is sufficient to produce inflammation, pus may form in the vein in a sufficient quantity to require an incision to enable it to escape.

Varices of long standing have generally been regarded as inveterate. In the only cases that have been cured, the result was obtained by imitating nature as nearly as possible; or, in other words, by producing a coagulation of the blood in the veins, which will always, for a time at least, afford relief. I have operated frequently, and in many cases the disease was permanently cured, but, occasionally, other veins became enlarged, which required a second operation. For many years I have used the toilet pin; and whenever blood followed the introduction of the pin it was removed, in consequence of the liability to phlebitis, and the dreadful consequences which result if allowed to remain. Dr. Venon, of Vervier, has recently cured varices of the legs by the application of a solution of the perchloride of iron. He directs it to be diluted with three parts of water, and strips composed of two or three thicknesses of lint should be placed over the enlarged vessels and secured by a bandage. According to this report the veins are obliterated in about two weeks.

Having read an extract from the London *Lancet* on this subject, and having always cases of this kind under my care, I determined to ascertain whether the treatment was reliable or not. The patient had varices of both legs. I performed Velpeau's operation with the pins on one leg, and used the perchloride on the other; three ounces of water were added to one of the perchloride; with this strips of lint three double were wet and applied over the enlarged veins, which were then covered with oiled silk and secured by a bandage. The dressing was renewed every day. In six or seven days the blood was coagulated in the small branches; and in one case the leg was cured in fifteen days, and was in a better condition than the one in which the pins were used. In the other case under treatment the small vessels were obliterated in ten days, and the internal saphena feels much more firm than before the treatment was commenced; and I think in a few days that the vessel will be entirely obliterated.

